# The Case in Favor of E-Cigarettes for Tobacco Harm Reduction

**DOI:** 10.3390/ijerph110606459

**Published:** 2014-06-20

**Authors:** Joel L. Nitzkin

**Affiliations:** JLN, MD Associates, Limited Liability Corporation (LLC), 4939 Chestnut Street, New Orleans, LA 70115, USA; E-Mail: jlnitzkin@gmail.com; Tel.: +1-504-899-7893; Fax: +1-504-899-7557

**Keywords:** e-cigarettes, ENDS devices, tobacco harm reduction, smoking cessation

## Abstract

A carefully structured Tobacco Harm Reduction (THR) initiative, with e-cigarettes as a prominent THR modality, added to current tobacco control programming, is the most feasible policy option likely to substantially reduce tobacco-attributable illness and death in the United States over the next 20 years. E-cigarettes and related vapor products are the most promising harm reduction modalities because of their acceptability to smokers. There are about 46 million smokers in the United States, and an estimated 480,000 deaths per year attributed to cigarette smoking. These numbers have been essentially stable since 2004. Currently recommended pharmaceutical smoking cessation protocols fail in about 90% of smokers who use them as directed, even under the best of study conditions, when results are measured at six to twelve months. E-cigarettes have not been attractive to non-smoking teens or adults. Limited numbers non-smokers have experimented with them, but hardly any have continued their use. The vast majority of e-cigarette use is by current smokers using them to cut down or quit cigarettes. E-cigarettes, even when used in no-smoking areas, pose no discernable risk to bystanders. Finally, addition of a THR component to current tobacco control programming will likely reduce costs by reducing the need for counseling and drugs.

## 1. Introduction

This paper makes the case for tobacco harm reduction (THR) with e-cigarettes as a prominent THR modality being the only feasible policy option with the potential to substantially reduce tobacco-attributable illness and death in the United States over the next twenty years, and do so without adversely effecting initiation or quit rates_._

Both of these papers are in support of the proposition that THR is the most feasible policy option likely to substantially reduce tobacco-attributable illness and death in the United States over the next 20 years. E-cigarettes and related vapor products, sometimes referred to as electronic nicotine delivery systems (ENDS) products are the most promising harm reduction modalities because of their acceptability to smokers.

It is important to note that THR is being proposed as an addition to, not a substitute for current tobacco control policy and programming. Retention and expansion of other tobacco control program elements will continue to be of value and offer the potential of yet further reductions in tobacco-attributable addiction, illness and death. Such program elements include but are not limited to health education, prohibition of marketing and sales to minors, packaging, labeling, warnings, and prohibition of smoking in multiple settings. 

The question as to whether or not FDA regulation of cigarettes and other tobacco products will further enhance or possibly harm addiction, illness and death rates is yet to be determined. FDA funding of health education initiatives and modification of combustible product labeling seems likely to have a minor effect on these outcomes. If, however, FDA regulation of lower risk alternative products is so stringent that it restricts their continuing evolution and marketing of e-cigarettes, it could have negative effects on future rates of illness and death while doing nothing to reduce continuing addiction of minors.

In practical terms, all of the potential benefits of THR accrue to smokers. Since no nicotine delivery product can be considered totally risk free, there is no potential benefit to non-smokers or persons who otherwise would not have initiated smoking. The potential harm of a THR initiative is the possibility that the initiative will attract to nicotine use a significant number of persons who otherwise would never have initiated smoking use. Some have even speculated that a THR initiative would likely do more harm than good because many of those newly attracted to nicotine use would then progress to cigarettes [1,2].

There are three major sets of issues to be considered in articulating the cases for and against THR and e-cigarettes as a harm reduction modality. The first is the safety of the product in terms of potential to cause serious illness in both users and bystanders. The second is the efficacy of the product in getting smokers to reduce or quit cigarettes. The third is the potential of the e-cigarette or other harm reduction modalities to recruit teens to nicotine use, and eventually to cigarettes.

The purpose of this paper is to set the stage for additional scientific study, policy dialogue and regulation development to confirm or deny current impressions and proceed with development of policies and regulations that would enable us to capture the public health benefits e-cigarettes can offer to smokers while not increasing, and likely decreasing teen initiation of tobacco use. 

There are about 46 million smokers in the United States, and an estimated 480,000 deaths per year attributed to cigarette smoking. Despite our best efforts, these numbers have been essentially stable since 2004 [3,4]. Reducing the number of smokers and numbers of deaths will require adding one or more new elements to current tobacco control programming.

The most recent Surgeon General Report [4] upped annual estimates of tobacco-attributable deaths in the United States from 443,000 to 480,000 per year, due to new research showing yet additional diseases to be attributable to cigarette smoking. All of the 480,000 estimated tobacco-attributable deaths each year in the USA are due to a single tobacco product—the machine-made cigarette [4]. Deaths from all other tobacco products are so low in number and so hard to distinguish from background that they are not tracked by American federal agencies. 

If the current flat trend continues, as it appears likely to do, an estimated 9,600,000 Americans will die of a cigarette-related illness over the next 20 years (480,000 deaths per year × 20 years). Since there is a 15–20 year delay between initiation of cigarette use and onset of potentially fatal tobacco-attributable cancer, heart, lung and other disease, and another 10–20 year delay from onset of illness to death—the vast majority of the 9.6 million deaths due to cigarette use in the USA over the next 20 years will be in current adult smokers who are now over 35 years of age. This means that reduction in teen smoking will not measurably reduce the numbers of deaths until 25–40 years from now.

The best we now have to offer current smokers is a set of pharmaceutical-based smoking cessation protocols that we know will fail about 90% of smokers who use them under the best of study conditions, with results measured at six to twelve months [5,6]. The flaws in the current “evidence-based” policies are fairly obvious. They do not satisfy the urge to smoke in the majority of smokers, the dose is too low, the duration of treatment too short and there is no built-in provision for self-reinforcement when the urge to smoke returns. 

### 1.1. Tobacco Harm Reduction (THR)

“Harm reduction” does not mean “harmless”. All of these products, including the pharmaceutical nicotine products pose more of a potential health risk than usually accepted in other consumer products. None are risk free. It is only in comparison to cigarettes that they can be considered very low risk. THR differs from smoking cessation medical therapy in that “therapy” implies a short-term (usually 12 weeks) course of medication, while THR implies use of the substitute product as long as the user feels the need for the product in question.

Tobacco harm reduction is envisioned in the United States as an educational initiative by which smokers who are unable or unwilling to quit are advised that they can lower their risk of a potentially fatal tobacco-attributable illness by 98% or better by switching to any one of the smokeless products now on the American market. These data are based on long-term epidemiological studies of Snus use in Sweden and on use of “smokeless tobacco” in the USA since the mid 1980’s [7,8]. Since e-cigarettes are basically a nicotine-only product with only the smallest traces of the carcinogens and other toxins found in smokeless tobacco product, e-cigarettes likely carry even less risk.

Several studies indicate, that, in addition to be less hazardous than cigarettes, the smokeless tobacco products currently available on the American and Scandinavian markets are also easier to quit [9,10]. One recent study shows a striking increase in quit attempts associated with the proliferation of electronic cigarettes [11].

According to Joel L. Nitzkin’s calculations [12], amended to reflect the new Surgeon General estimate, a modestly successful THR initiative, if added to current tobacco control programming would likely save the lives of 1.5 to 4.8 million of these Americans, with the numbers dependent on the rate of switching from cigarettes to lower risk smoke-free products. In Year 20 of such an intervention, again, depending on the actual switch rates, numbers of smokers and numbers of deaths would be down 30% to 80%, from current year estimates.

There is no other feasible tobacco control policy that has the potential to secure public health benefits of this magnitude. It would consist of simply telling the truth to the American public about the differences in risk, comparing cigarettes to lower risk smoke-free options. There would be no drugs to buy and no additional health education programming. THR would be an addition to, not a replacement for current tobacco control programming. Prohibition of sales to minors, strict regulation of manufacture and marketing, clean indoor air regulations, tax policy and control of contraband would remain in place, and hopefully be strengthened.

The health education component of the THR initiative would be of very low cost or free to the taxpayer as much, if not all the cost would be carried by the manufacturers and vendors. There would be some additional cost to evaluate the impact of the THR initiative. It seems likely that this cost would be borne by licensure fees paid by manufacturers to the FDA.

## 2. Methods

The data and narrative reported herein are based on published literature, media reports, blogs and internet sites supplemented with policy analysis.

## 3. Results and Discussion

### 3.1. E-cigarettes/Nicotine Vaporizers

Nicotine vaporizers, including e-cigarettes and related products, show substantial promise as a vehicle for THR. Skyrocketing sales of e-cigarettes, consumer advocacy for these products, and a flood of new scientific papers relating to these products suggest the possibility that e-cigarettes may be the greatest advance in reducing tobacco-attributable illness and death in decades. Moreover, progress to date has been accomplished at no cost to the taxpayer and with little or no recruitment of teen non-smokers [11,13,14,15,16,17,18].

### 3.2. Step-down in Risk from Cigarette Smoking to E-cigarette Vapor

#### 3.2.1. Cigarettes

The Centers for Disease Control and Prevention estimates there are 480,000 tobacco-related deaths annually in the United States and all are from cigarette use [3,4]. Deaths from other forms to tobacco are so small and so hard to estimate that they are not estimated or tracked by the CDC.

Tobacco cigarettes are the most hazardous and addictive of tobacco products, and the product most attractive to teens. There was no pandemic of tobacco-related addiction, illness and death until the advent of the machine-made cigarette.

#### 3.2.2. Environmental Tobacco Smoke (ETS)

Tobacco smoke is a witch’s brew of toxic chemical substances from the incomplete combustion of tobacco. The main component is carbon monoxide, but it also includes other gasses and tarry particulate residue containing most of the nicotine and the worst of the carcinogens [19].

About 85% of environmental tobacco smoke (ETS), commonly called “second hand smoke”, is the smoke that curls off the end of a cigarette when no-one is puffing on it. Solid particles make up about 10% of the smoke, including the tar and most of the nicotine [20]. The mainstream smoke exhaled by the smoker includes only what is left after much of what was inhaled is absorbed by the smoker.

ETS increases the risk of lung cancer and other cancers; heart and lung disease; the risk of low birth weight; and is suspected of increasing the risk of birth defects. CDC estimates that approximately 50,000 non-smokers die in the United States from exposure to ETS [3]. In addition, ETS is known to irritate the eyes, throat, and respiratory mucous membranes [20]. 

#### 3.2.3. Smokeless Tobacco Products on the U.S. Market

The smokeless tobacco products which have been on the U.S. market since the 1980s are estimated to pose a risk of potentially fatal illness less than 2% the risk posed by cigarettes [7]. 

E-cigarettes are one of a number of smoke-free tobacco/nicotine alternatives to cigarettes that can reduce the risk of tobacco-attributable illness and death by 98% or better, while satisfying the smoker’s urge for nicotine. These include chewing tobacco; snus and other snuff products; dissolvables (sticks, strips and orbs), and e-cigarettes. Options also include use of pharmaceutical nicotine replacement therapy (NRT) products such as patches, gum, lozenges, and inhalers on a long-term basis in a harm-reduction mode.

#### 3.2.4. E-cigarettes and Other ENDS Devices

E-cigarettes and related devices are currently the most promising THR option. These metal or plastic tubes use a battery, heating element and small amount of nicotine-containing fluid to give smokers nicotine without the high concentration of thousands of other toxic chemicals that exist in cigarette smoke. These devices also emulate the cigarette-handling ritual and the feel of cigarette smoke in the mouth and throat. 

These devices are unique in the U.S. marketplace in that they are the only smoke-free tobacco products that do not carry mandated warnings about cancer or other diseases. They are also unique in terms of their skyrocketing sales. Bonnie Herzog, Wells Fargo’s managing director for beverage, tobacco and convenience store research, predicted in January 2013 that “consumption of e-cigs may overtake traditional cigarettes in the next decade [21]”. At that time, e-cigarette sales were projected at $1 billion for 2013. In mid-September, Herzog upped her projection to “around $2 billion by the end of the year and up to $10 billion by 2017”, adding that she expects electronic products would overtake tobacco cigarettes within the next decade [22].

#### 3.2.5. Environmental E-cigarette Vapor

The e-cigarette vapor inhaled by users consists mainly of water, propylene glycol and glycerin, with small amounts of nicotine and flavoring. There is no carbon monoxide, no tar, and no products of combustion. There is no side-stream smoke or vapor. Propylene glycol and glycerin are generally recognized as safe. Propylene glycol has been used as the propellant in asthma inhalers and is the main ingredient in theatrical fog. There is a question about the safety of intense of pulmonary propylene glycol exposure from e-cigarettes. This is a question in need of additional research. 

E-cigarettes have no products of combustion. Nothing curls off the end of an e-cigarette when no one is puffing on it. The mainstream vapor exhaled by the user includes only the tiniest traces of chemical contaminants.

E-cigarette vapor, as exhaled by the e-cigarette user, poses no significant risk to bystanders [23]. A number of studies have been published dealing with the concentration of organic chemicals in exhaled e-cigarette vapor. Basically, these studies show that when the e-cigarette user exhales into a glass tube or similar container, trace quantities of a variety of organic chemicals can be detected, but, when in an 8 cubic meter test chamber or similar room, for a half hour or more, e-cigarette use does not measurably increase the trace quantities of these chemical substances above background levels, while cigarettes cause dramatic rapid increases [24,25,26,27].

An October 2012 study published in *Inhalation Toxicology* found that, for all byproducts measured, e-cigarettes produced very small exposures relative to tobacco cigarettes, indicating no apparent risk to human health from e-cigarette emissions [28]. Further research presented to Europe’s Society for Research on Nicotine and Tobacco compared total organic carbons in a test chamber five hours after smoking or vaping, finding no detectable levels of acrolein, toluene, xylene and polycyclic aromatic hydrocarbons (PAH) in the e-cigarette vapor compared to high levels in the cigarette chamber [26]. 

Perhaps the most interesting findings have been studies showing that persons not using any form of tobacco routinely exhale trace amounts of acetone, ethane, pentane and isoprene and other endogenous volatile organic compounds [29,30,31,32].

In tests comparing the effects of e-cigarette vapor to cigarette smoke on cell cultures of myocardial cells, the vapor had minimal impact on the cells, while the smoke killed almost all of them [33].

#### 3.2.6. Pharmaceutical Nicotine Replacement Therapy Products

Use of pharmaceutical nicotine replacement therapy products, such as Nicorette, Commit, and others, is perceived by public health authorities to pose no risk of tobacco-attributable illness and death, despite the presence of many of the same trace contaminants that exist in e-cigarettes. 

Nitrosamine levels in e-cigarettes and related devices have been found to be similar to the levels in Nicorette gum and NicoDerm patches, but less than one-hundredth to one-thousandth the level in a wide range of smokeless tobacco and cigarette products [34]. 

The major problem with current reliance on pharmaceutical nicotine replacement therapy “evidence-based” protocols is that they fail about 90% of the smokers who use them, even under the best of study circumstances [5]. The need to add a THR element to current tobacco control programming is largely based on experience to-date that large numbers of smokers who are unable or unwilling to quit using the pharmaceutical products. A smoker can eliminate almost all exposure to the many toxins in cigarettes by switching to e-cigarettes [23,28].

### 3.3. Lack of Attractiveness of E-cigarettes to Non-smoking Teens and Adults

Two recently published studies conducted by public health non-profits—one in the U.S. and the other in the United Kingdom show that teens are very aware of e-cigarettes, but researchers were unable to find even a single non-smoking teen who had taken them up. One study published online in the *Journal of Environmental and Public Health* and co-authored by Dr. Jonathan Winickoff, chairman of the American Academy of Pediatrics’ Tobacco Consortium, was able to find only six nonsmokers who had ever used e-cigarettes in a national survey of 3240 adults, including 1802 non-smokers [35]. 

The American study noted above did not include children, but a concurrent study in Great Britain did. A second study from Action on Smoking and Health (ASH-UK), also contradicts the anti-smoking groups’ contention that electronic cigarettes appeal to nonsmokers, especially youth. ASH-UK were unable to find a single nonsmoker in Great Britain—either youth or adult—who regularly uses electronic cigarettes [14]. The group’s study was based on a survey of 12,171 adults and. The ASH-UK survey of 2178 children ages 11–18 in February and March of 2013 study found awareness of electronic cigarettes was 67% among those between the ages of 11 and 18 and 83% among those between the ages of 16 and 18. Nevertheless, it found that, among young people who had never smoked, “0% report continued e-cigarette use and 0% expect to try an e-cigarette soon”. They surveyed 12,171 adults. The study also found that, among adults who had never smoked, none reported current electronic cigarette use [14]. 

In early September 2013, CDC published a study showing that e-cigarette use among middle and high school students had doubled from 2011 to 2012 [36]. In response to these data, CDC Director Thomas Frieden proclaimed: “The increased use of e-cigarettes by teens is deeply troubling. Many teens who start with e-cigarettes may be condemned to struggling with a lifelong addiction to nicotine and conventional cigarettes”. A similar sentiment was restated in a 2014 re-analysis of this same CDC surveillance data [37]. Despite the hype surrounding these publications, the survey data equally support the opposite conclusion, that e-cigarettes are not attractive to teens and that e-cigarettes are likely to result in future declines in teen smoking [18,38,39]. Most importantly, in the CDC data, the dramatic increase in e-cigarette use by middle and high school students co-occurred with a further reduction in teen smoking [36].

The surveys noted above show that the currently unregulated e-cigarettes attract almost no non-smokers. This, in turn, suggests that it should be possible to endorse these products to smokers without fear that large numbers of teen and other non-smokers will be attracted by such endorsement.

### 3.4. Relative Addictiveness of Different Classes of Tobacco/Nicotine Product

On 14 December 2013, Karl Fagerstrom posted a well referenced essay entitled “Dependence on Tobacco and Nicotine” on the Nicotine Science and Policy website [9]. In this essay he makes a very strong case for their being a “continuum of dependence” in which cigarettes foster the strongest dependence, NRT pharmaceuticals the least, with smokeless products, e-cigarettes and other products in-between. Elements relating to the strength of the dependence include other chemical substances in cigarette smoke, habituation to the cigarette-handling ritual and social and psychological factors. The practical implication of this essay is to the effect that when a smoker switches to a lower risk smokeless product or ENDS device, not only does he or she dramatically reduce future risk of potentially fatal tobacco-attributable illness, he or she is switching to a product that should be easier to quit than cigarettes.

### 3.5. Contraband

One of the major problems with taxation of cigarettes and possible future steps to make cigarettes less desirable to smokers by eliminating menthol or reducing nicotine content is contraband. Making the legal product more expensive or less desirable is virtually guaranteed to increase sales of illicit and contraband product. This response, in turn, has the capability to totally eliminate any public health benefit from the intervention in question. The Mackinac Center for Public Policy recently estimated smuggling rates in 47 of the 48 contiguous U.S. states, based on 2012 data. New York, with the highest cigarette tax had the highest inbound cigarette smuggling rate with an estimated 56.9% of total cigarettes consumed being smuggled. This was followed by 51.5% for Arizona, 48.1% for New Mexico, 48% for Washington, and 34.6% for Wisconsin [40].

A major advantage of THR is that, by making all cigarettes less desirable to smokers, the benefits of a THR initiative cannot be neutralized by increased sale of contraband cigarettes. 

### 3.6. Consumption of Cigarettes by Mental Health Patients

Adults who suffer from depression are twice as likely to smoke and also smoke more heavily than other adults, according to a survey from the National Center for Health Statistics [41]. Persons with a mental disorder in the month prior to a national comorbidity survey consumed approximately 44.3% of the cigarettes smoked by this nationally representative sample [42]. In addition, mental health patients have a harder time quitting than others [43].

A number of studies demonstrate that depressed patients and those with bipolar disorder and/or schizophrenia find self-administered nicotine to be highly beneficial. It helps them get through the day in emotional balance and with substantially fewer side effects than the usually prescribed medications [44,45,46]. Thus, total elimination of non-prescription nicotine delivery products, as desired by many anti-tobacco advocates would be harmful to many mental health patients. 

## 4. Conclusions

The time has come to add a tobacco harm reduction (THR) component to current tobacco control programming, with e-cigarettes and related vapor devices as primary THR modalities. THR is the only feasible policy option likely to substantially reduce tobacco-attributable illness and death in the United States over the next 20 years. THR, with e-cigarettes can help decrease teen initiation of nicotine use, and help increase quit rates. THR is especially important for smokers who are unable or unwilling to quit because of the benefit they secure from non-prescription self-administered nicotine.

## References

[1] Siegel M.B. CDC Director Apparently Fabricating More “Scientific Evidence” to Demonize Electronic Cigarettes. http://www.tobaccoanalysis.blogspot.com/2014/05/cdc-director-apparently-fabricating.html.

[2] Grana R., Benowitz N., Glantz S.A. (2014). E-cigarettes: A scientific review. Circulation.

[3] Centers for Disease Control and Prevention Tobacco-related Mortality, in CDC Fact Sheet-tobacco Related Mortality Smoking and Tobacco Use. http://www.cdc.gov/tobacco/data_statistics/fact_sheets/health_effects/tobacco_related_mortality/index.htm.

[4] (2014). The Health Consequences of Smoking—50 Years of Progress.

[5] Moore D., Aveyard P., Connock M., Wang D., Fry-Smith A., Barton P. Effectiveness and Safety of Nicotine Replacement Therapy Assisted Reduction to Stop Smoking: Systematic Review and Meta-analysis. http://www.bmj.com/cgi/content/full/338/apr02_3/b1024.

[6] Zhu S.H., Lee M., Zhuang Y., Garnst A., Wolfson T. (2012). Interventions to increase smoking cessation at the population level: How much progress has been made in the last two decades?. Tob. Control.

[7] Rodu B. (2011). The scientific foundation for tobacco harm reduction, 2006–2011. Harm Reduct. J..

[8] Lee P. (2013). Epidemiologic evidence relating snus to health—An updated review based on recent publications. Harm Reduct. J..

[9] Fagerstrom K. Dependence on Tobacco and Nicotine,in Nicotine Science and Policy. http://nicotinepolicy.net/karl-fagerstrom/520-dependence-on-tobacco-and-nicotine.

[10] Zhu S.H., Wang J.B., Hartman A., Zhuang Y., Gamst A., Gibson J.T., Gillijam H., Galanti M.R. (2009). Quitting cigarettes completely or switching to smokeless tobacco: Do U.S. data replicate the Swedish results?. Tob. Control.

[11] Siegel M. Latest Data from UK Show a Striking Increase in Quit Attempts Associated with Proliferation of Electronic Cigarettes. http://tobaccoanalysis.blogspot.com/2013/10/latest-data-from-uk-show-striking.htm.

[12] Nitzkin J.L. (2010). Tobacco harm reduction: 20 year projections of smoking prevalence and smoking-related deaths in USA.

[13] Centers for Disease Control and Prevention (2012). National Youth Tobacco Survey. http://www.cdc.gov/tobacco/data_statistics/surveys/nyts/.

[14] (2013). Fact Sheet on the Use of E-cigarettes in Great Britain among Adults and Young People.

[15] Siegel M. CDC Director and Prominent Anti-smoking Researcher Appear to be Fabricating Scientific Evidence to Oppose Electronic Cigarettes. http://www.tobaccoanalysis.blogspot.com/2013/09/cdc-director-and-prominent-anti-smoking.html.

[16] Siegel M. Electronic Cigarette Experimentation Increases among Youth,but Use among Non-smokers Remains Low and Regular Use Rates Are Still Unknown. http://tobaccoanalysis.blogspot.com/2013/09/electronic-cigarette-experimentation.html.

[17] Action on Smoking and Health Use of Electronic Cigarettes in Great Britain. http://www.ash.org.uk/files/documents/ASH_891.pdf.

[18] Siegel M. If Electronic Cigarettes Are a Gateway to Smoking,Then Why Were Youth Smoking Rates at an All-time Low in 2013?. http://tobaccoanalysis.blogspot.com/2014/01/if-electronic-cigarettes-are-gateway-to.html.

[19] Martin T. Environmental Tobacco Smoke,in About.Com: Smoking Cessation. http://quitsmoking.about.com/cs/secondhandsmoke/g/ETS.htm.

[20] Fact Sheet: Environmental Tobacco Smoke: A Toxic Air Contaminant. http://www.arb.ca.gov/toxics/ets/factsheetets.pdf.

[21] Sanburn J. Can Electronic Cigarettes Challenge Big Tobacco?. http://business.time.com/2013/01/08/can-electronic-cigarettes-challenge-big-tobacco/.

[22] Ranks V. Analyst Confirms Projection That E-cigarettes Will Overtake Traditional Cigarettes in the Next Decade. http://vaperanks.com/analyst-confirms-projection-that-e-cigarettes-will-overtake-traditional-cigarettes-in-the-next-decade/#sthash.tYRn3D5q.dpuf.

[23] Burstyn I. Peering through the Mist: What Does the Chemistry of Contaminants in Electronic Cigarettes Tell Us about Health Risks?. http://publichealth.drexel.edu/SiteData/docs/ms08/f90349264250e603/ms08.pdf.

[24] Schripp T., Markewitz D., Uhde E., Salthammer T. (2013). Does e-cigarette consumption cause passive vaping?. Indoor Air.

[25] Czogala J., Goniewicz M., Fidelus B., Zielinska-Danch W., Travers M., Sobczak A. (2014). Secondhand exposure to vapors from electronic cigarettes. Nicotine Tob. Res..

[26] Romagna G., Zabarini L., Barbiero L., Bocchietto E., Todeschi S., Caravati E., Voster D., Farsalinos K. Characterization of Chemicals Released to the environment by Electronic Cigarettes Use (ClearStream-AIR Project): Is Passive Vaping a Reality?. Proceedings of 14th Annual Meeting of the Society for Research on Nicotine and Tobacco.

[27] Rodu B. Do E-cigarettes Cause Passive Vaping?. http://rodutobaccotruth.blogspot.com/2013/12/do-e-cigarettes-cause-passive-vaping.html.

[28] McAuley T., Hopke P., Zhao J., Babaian S. (2012). Comparison of the effects of e-cigarette vapor and cigarette smoke on indoor air quality. Inhal. Toxicol..

[29] Larstad M., Toren K., Blake B., Olin A.C. (2007). Determination of ethane, pentane and isoprene in exhaled air—Effects of breath-holding, flow rate and purified air. Acta Physiol..

[30] Smith D., Spanel P., Enderby B., Lenney W., Turner C., Davies J. Isoprene Levels in the Exhaled Breath of 200 Healthy Pupils within the Age Range 7–18 Years Studied Using SIFT-MS. http://www.iopscience.iop.org/1752-7163/4/1/017101.

[31] King J., Koc H., Unterkofler K., Mochalski P., Kupferthaler A.T.G., Teschl S., Hinterhuber H., Amann A. (2010). Physiological modeling of isoprene dynamics in exhaled breath. J. Theor. Biol..

[32] King J., Kupferthaler A., Frauscher B., Hackner H., Unterkofler K., Teschl G., Hinterhuber H., Amann A., Hogl B. Measurement of Endogenous Acetone and Isoprene in Exhaled Breath during Sleep. http://iopscience.iop.org/0967-3334/33/3/413.

[33] Farsalinos K. Research on Safety of Electronic Cigarettes, Overview of Existing Research on E-cigarettes and Other Alternatives. Proceedings of 98th Annual Meeting of the Tobacco Merchants Association.

[34] Cahn Z., Siegel M. (2011). Electronic cigarettes as a harm reduction strategy for tobacco control: A step forward or a repeat of past mistakes?. J. Public Health Policy.

[35] McMillen R., Maduka J., Winickoff J. (2012). Use of emerging tobacco products in the United States. J. Environ. Public Health.

[36] Centers for Disease Control and Prevention Notes from the Field: Electronic Cigarette Use among Middle and High School Students—United States, 2011–2012. http://www.cdc.gov/mmwr/preview/mmwrhtml/mm6235a6.htm.

[37] Dutra L., Glantz S. (2014). Electronic cigarettes and conventional cigarette use among U.S. adolescents: A cross-sectional study. JAMA Pediatrics.

[38] Siegel M. Glantz Press Release is Dishonest with Public. Authors Appear to Be Intentionally Lying to Misldead the Media and the Public. http://www.tobaccoanalysis.blogspot.com/2014/03/glantz-press-release-isdishonest-with.html.

[39] Phillips C. Stanton Glantz is Such a Liar That Even the ACS Balks: His Latest Ecig Gateway “Study”, in Anti-THR Lies and Related Topics. http://antithrlies.com/2014/03/07/stanton-glantz-is-such-a-liar-that-even-the-acs-balks/.

[40] Tobacco Merchant’s Association TMA Executive Summary. http://www.tma.org/tmalive/Upload/ES1412.pdf.

[41] Depressed Patients Smoke More: Study. http://phys.org/print190471659.html.

[42] Lasser K., Boyd J., Woolhandler S., Himmelstein D., McCormick D., Bor D. (2000). Smoking and mental illness: A population-based prevalence study. JAMA.

[43] LeCook B., Wayne G., Kafali E., Liu Z., Flores S. (2014). Trends in smoking among adults with mental illness and association between mental health treatment and smoking cessation. JAMA.

[44] Kumari V., Postma P. (2005). Nicotine use in schizophrenia: The self medication hypotheses. Neurosci. Biobehav. Rev..

[45] Sacco K., Termine A., Seyal A., Dudas M., Vessicchio J., Krishnan-Sarin S., Jatlow P.I., Wexler B.E., George T.P. (2005). Effects of cigarette smoking on spatial working memory and attentional deficits in schizophrenia: Involvement of nicotinic receptor mechanisms. Arch. Gen. Psychiat..

[46] Wing V., Bacher I., Sacco K., George T. (2011). Neuropsychological performance in patients with schizophrenia and controls as a function of cigarette smoking status. Psychiat. Res..

